# A High Throughput Micro-Chamber Array Device for Single Cell Clonal Cultivation and Tumor Heterogeneity Analysis

**DOI:** 10.1038/srep11937

**Published:** 2015-07-07

**Authors:** Feng-Min Shen, Lian Zhu, Heng Ye, Yu-Jun Yang, Dai-Wen Pang, Zhi-Ling Zhang

**Affiliations:** 1 Key Laboratory of Analytical Chemistry for Biology and Medicine (Ministry of Education), College of Chemistry and Molecular Sciences, Wuhan University, Wuhan, 430072, P. R. China

## Abstract

Recently, single cell cloning techniques have been gradually developed benefited from their important roles in monoclonal antibody screening, tumor heterogeneity research fields, etc. In this study, we developed a high throughput device containing 1400 lateral chambers to efficiently isolate single cells and carry out long-term single cell clonal cultivation as well as tumor heterogeneity studies. Most of the isolated single cells could proliferate normally nearly as long as three weeks and hundreds of clones could be formed once with one device, which made it possible to study tumor heterogeneity at single cell level. The device was further used to examine tumor heterogeneity such as morphology, growth rate, anti-cancer drug tolerance as well as adenosine triphosphate-binding cassette (ABC) transporter ABCG2 protein expression level. Except for the single cell isolation and tumor heterogeneity studies, the device is expected to be used as an excellent platform for drug screening, tumor biomarker discovering and tumor metastasis assay.

Heterogeneity widely exists among tumor cells. To date, tumor heterogeneity^1^,^2^ has become one of the most attractively scientific topics among tumor related studies attributed to its potential importance on tumor invasion, metastasis, clinical treatments and predictions of patients survivals and so on^3^,^4^,^5^. Traditional studies on tumor heterogeneity were generally confined to bulk cell population, which merely revealed average cell behaviors and was prone to miss some key information useful for tumor heterogeneity researches and early clinical diagnosis. Single cell cloning techniques became an ideal platform for studying tumor heterogeneity. The relationships between single cell clonal evolution and tumor heterogeneity have been studied for several decades. Series of events occurred during the fission and clonal proliferation of single cells, through which some of the cells survived and acquired preponderant characteristics over other cells, then heterogeneity happened^6^. Single cell isolation and clonal cultivation pave ways for tumor heterogeneity researches^7^,^8^,^9^,^10^,^11^,^12^,^13^,^14^,^15^. Traditional single cell cloning techniques including limiting dilution cloning, feeder layer cloning, agar cloning have made great contribution to tumor cell heterogeneity studies. However, some disadvantages, such as time-consuming, complicated experimental procedures, lower single cell isolation and cloning efficiency, have greatly restricted their further applications and brought too much uncertainty to heterogeneity researches. There is an urgent requirement of high throughput single cell isolation and clonal cultivation techniques for single cell-based tumor heterogeneity studies.

Microfluidic techniques attributed to their unique advantages, such as flexible designing of the specific structure, high throughput capacity and parallel size scale with common organisms especially cells, which have positively promoted their developments for advanced cell biological studies^16^,^17^,^18^, have represented one of the most excellent platforms for high throughput single cell analysis nowadays. Some relative approaches have been established based on microfluidic devices. The published reports were summarized into two main aspects namely spatial structure constraint dominated models^19^,^20^,^21^,^22^ and droplet package isolation strategies^23^,^24^,^25^. Carlo *et al.* took use of a U-shaped hydrodynamic trapping structure to capture single cells and analyze single cell division^19^. Matsumura *et al.* designed a device to fulfill long-term clonal culture and trace the clone growth at single cell level^22^. Aside from the physical structures mentioned above, several other similar models have also emerged^20^,^21^. Although some of these models provided novel approaches for single cell isolation, some of the designs based on physical constraints inevitably exposed the isolated single cells under hydrodynamic shear stress which had detrimental impacts on isolated cells and made the relevant device not apt for long-term clonal cultivation. Some of designs needed additional surface modification, which made the fabrication process laborious and time consuming. Last but not least, no more molecular level studies were conducted after clonal cultivation among the studies above. Besides, only few of the designs could achieve long-term clonal cultivation. Droplet-based single cell isolation devices have been applied for probing cellular heterogeneity and antibody screening without clonal cultivation^26^,^27^. The droplet-based devices guaranteed high single cell isolation efficiency and eased cross-contamination. However, due to insufficient supplements of fresh medium, narrow space and accumulated poisonous metabolites, continuous cultivation of the isolated single cells within the mono-dispersed droplets was difficult, which impeded their applications in cell heterogeneity researches. Few reports on single cell clone and heterogeneity studies were published. Recently, Guan *et al.* established a micro-collagen gel array for 3D single cell clonal cultivation and heterogeneity studies at single cell level with a microfluidic device^28^.

Based on the recent researches as well as our studies, there are three key points essential for long-term single cell clonal cultivation in microfluidic devices: 1) adequate space for single cell long-term clonal proliferation; 2) dynamic perfusion system for fresh nutrient supplement and poisonous metabolite excretion; 3) minimum negative impacts of hydrodynamic shear stress on isolated cells. To meet the needs above and improve the practicability of device, herein, we designed a high throughput single cell clonal cultivation array with 1400 lateral square chambers symmetrically connected to the main channels. The simulation result showed the designed device greatly decreased the negative effect of hydrodynamic shear stress within the lateral cell culture chambers. Moreover, the isolated single cells could proliferate normally and grew for nearly as long as three weeks to form single cell clones, which made tumor heterogeneity studies based on single cell level available. After systematic optimization, sample injection could be finished in less than 3 minutes and hundreds of clones can be obtained once. We found two sub-types of cells among all the clones were formed. Tumor cell heterogeneity on cell morphology, growth rate, drug sensitivity and ABCG2 protein expression difference have been conducted at single cell clone level on the device.

## Results

### Configuration of the device

**Figure 1:**
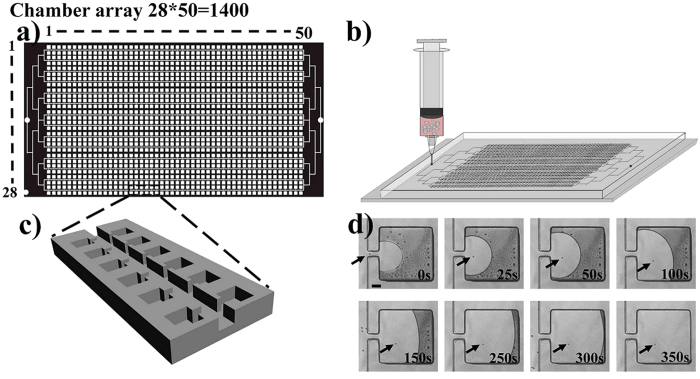
Schematic view of the device and cell suspension injection, (**a**) the structure of the designed chip. (**b**) cell suspension injection into the chip. (**c**) enlarged view of the chamber array. (**d**) dynamic course of single cell self-loading into the lateral chamber. The arrows refer to a single cell. Scale bar in Fig. 1d is 100 μm.

The designed device contains 1400 lateral square culture chambers with 500 micrometers per side connected to 100 micrometers wide perfusion channels through 30 micrometers wide lateral entrances as shown in Figs 1a,c. In this work, 30 micrometers and 500 micrometers were chosen as the optimal size of lateral entrance and chamber respectively for single cell self-loading and long-term clonal cultivation. When the size of chamber is larger than 500 micrometers, to capture the whole culture chamber area with one image becomes impossible under a 10 × objective, which leads to a labor-intensive observation process. While smaller than 500 micrometers, long-term single cell clonal cultivation will be partially restricted because of limited space of the culture chambers. As shown in Figs 1b,d, when the cell suspension was injected into the device, it was possible to load a single cell into a lateral culture chamber, the corresponding dynamic course was displayed in Fig. S1 Movie, Electronic Supplementary Information. The capacious micro-channel and lateral micro-chamber were designed to protect isolated single cells from detrimental effect of hydrodynamic shear stress^29^,^30^,^31^. COMSOL Multiphysics 4.3 simulation result showed that the surface velocity within the culture chambers was extremely low (as shown in Fig. S1,), which indicated that the impact of hydrodynamic shear stress was negligible, due to a positive correlation between hydrodynamic shear stress and flow rate^32^. Besides, dynamic perfusion system is essential for fresh medium supplement and poisonous metabolites discharging based on the effective diffusion, which is important for long-term clonal cultivation. The diffusion process between the main channels and lateral chambers was confirmed by the diffusion experiments of red ink into and out of the lateral chambers (as shown in Fig. S2a,b). The designed structure and dynamic perfusion system guaranteed both high throughput single cell isolation efficiency and long-term clonal cultivation.

### High throughput and long-term single cell clonal cultivation

**Figure 2:**
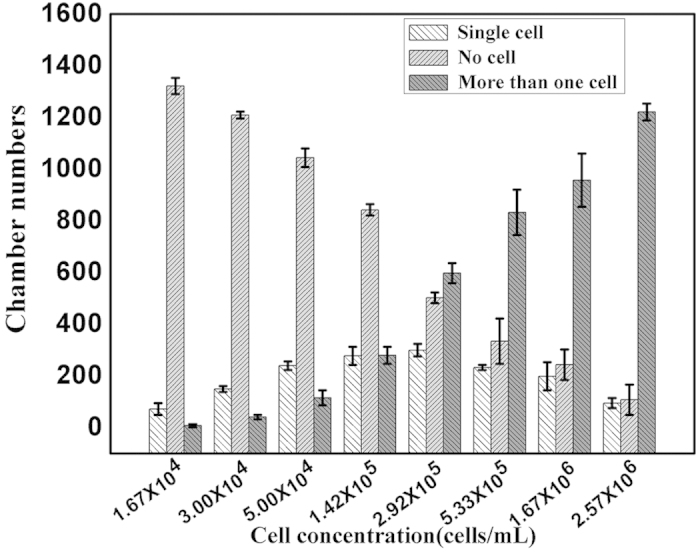
Relationship between cell suspension concentrations and numbers of chamber containing one cell, none cell and more than one cell. White histograms refer to numbers of chamber with one cell, the gray histograms refer to numbers of empty chamber, the black histograms refer to chamber numbers with more than one cell.

In order to improve single cell isolation efficiency, series of condition optimizations were conducted. Firstly, we examined the effect of cell suspension concentration on single cell isolation efficiency, different concentrations such as 1.67 × 10^4^, 3.00 × 10^4^, 5.00 × 10^4^, 1.42 × 10^5^, 2.92 × 10^5^, 5.33 × 10^5^, 1.67 × 10^6^ and 2.57 × 10^6^ cells/mL were respectively examined as shown in Fig. 2. The results clearly demonstrated that the numbers of single cell chamber firstly increased with the increased cell suspension concentration, then decreased when the concentration reached to 2.92 × 10^5^ cells/mL. The numbers of chamber without cell decreased with the increased cell suspension concentration during the whole range. Besides, the number of chambers included more than one cells was also increased with the increased cell suspension concentration. In consideration of the effect of the cell suspension concentration on the following single cell clonal cultivation, we here chose 3 × 10^5^ cells/mL as the optimum concentration throughout the studies, about 598 lateral chambers included more than one cell under the optimized cell suspension concentration as showed in Fig. 2.

Then, relative parameters, injection flow rate and vacuumization time, were also examined (the results were shown in Fig. S3a,b). Fig. S4a showed that flow rate hardly affected single cell isolation efficiency in the range of 50 to 1000 μL/min. In order to improve efficiency, manual injection was used to load cell suspension into the device. As we known, vacuum degree inside the device also contributed to the diffusion of the cells into the lateral chambers. According to the data showed in Fig. S4b, half an hour was enough for vacuumization. Finally, high throughput single cell isolation and long-term cell clonal cultivation were undertaken based on the optimized conditions. About three hundreds single cells can be obtained with one device once, which is unimaginable in traditional single cell cloning techniques^7^,^8^,^9^,^10^,^11^.

**Figure 3:**
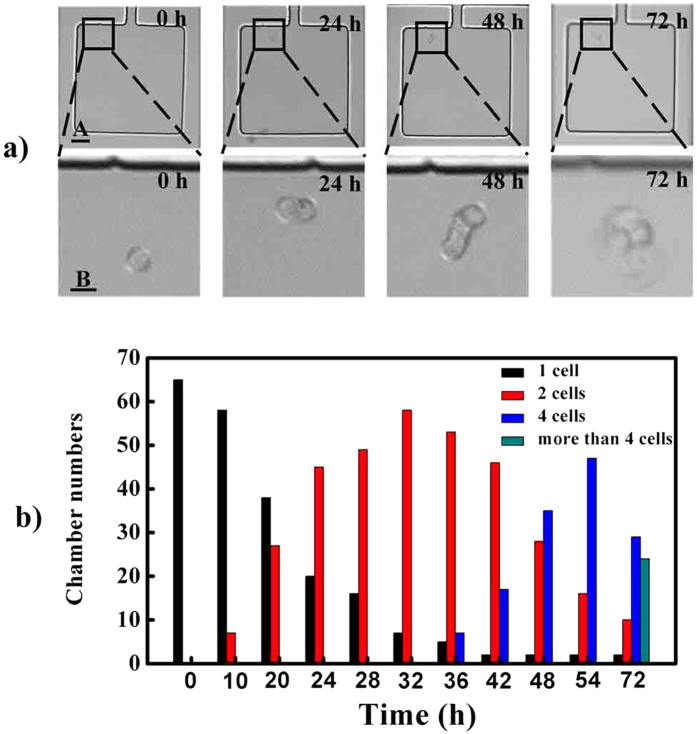
Single cell clonal proliferation within 72 h. (**a**) images of single cell proliferation from 0 h to 72 h. (**b**) dynamic statistics of single cell proliferation. Scale bars of A, B in Fig. 3a are 50 and 100 μm respectively.

On-chip real-time monitoring of single cell proliferation can be accomplished under a confocal microscope equipped with a CO_2_ online culture system. When loaded into the lateral chambers, single cells could gradually attach on the glass substrate and grew. Figure 3a showed that the isolated single cell proliferation could be realized in the lateral chamber. The single cell divided into two cells at 24 h, four cells at 48 h and grew to be a cell cluster at 72 h.

Statistically, a fraction of all the isolated single cells in the designed device, about 10%, divided into two cells at 10 h. From 10 h to 32 h, more and more single cells began to split into two cells. At 36 h, a fraction of single cells, about 10%, split into four cells, and at 72 h, some of single cells split into more than four cells as shown in Fig. 3b. However, during this period, nearly 3% still kept quiescent without the ability to proliferate to form clones. The proliferation difference of isolated single cells may be related to cell cycle or their intrinsic heterogeneity^33^.

With the aid of lateral chamber designing, and dynamic perfusion system, most of the isolated single cells can proliferate for approximate as long as three weeks (Fig. S4). Usually, in the traditional single cell cloning techniques, only 4% clone formation ratio could be obtained in a 96-wells plate with a serial of complicated and labor-intensive manipulations. Besides, the device was proved to be apt for suspension tumor cell clonal cultivation as shown in Fig. S5, Fig. S6. The designed microfluidic device could isolate single cells into lateral culture chambers under simple manual injection. And combined with single cell cultivation, hundreds of single cell clones could be obtained in one device, which greatly benefits tumor heterogeneity study at single cell level.

### Tumor heterogeneity studies on morphology, growth rate, clone sensitivity to indomethacin and ABCG2 expression

**Figure 4:**
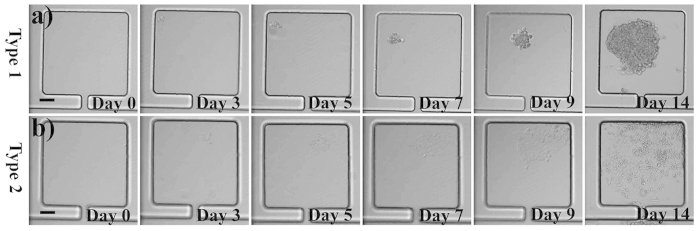
Clones proliferation and morphologies distribution in chambers (days 0–14) starting from a single MHCC 97L cell. (a) type 1 MHCC 97L, b) type 2 MHCC 97L. Scale bars in Figs 4a,b are both 100 μm.

Tumor heterogeneity includes the following aspects namely size difference, different morphologies, relative protein expression level, growth rate and sensitivity to anti-cancer drug and so on^3^. The text above had displayed that hundreds of clones could be formed in one device once. Among these clones, two apparent cell morphologies were discovered. Most of the clones grew adherently as multilayer and aggregates, we called them type 1 MHCC 97L (Fig. 4a), the other clone grew adherently as a uniform monolayer, and we called them type 2 MHCC 97L (Fig. 4b). Based on the statistics, the percentages of the type 1 and type 2 clones are respectively 97% and 3% (Fig. S7).

**Figure 5:**
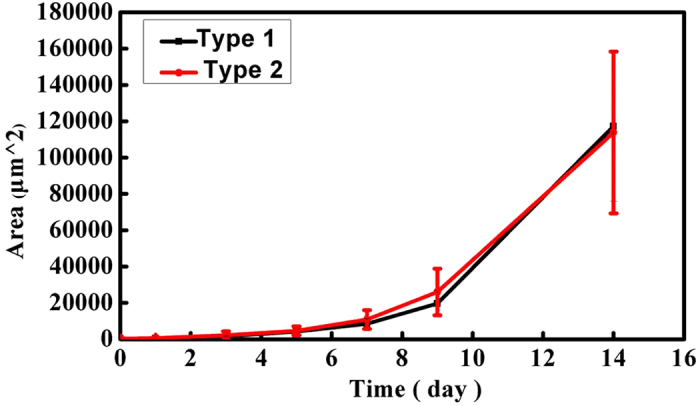
Growth curves of both type 1 and type 2 MHCC 97L cells in chip chambers.

Some reports pointed out that growth rate, sensitivity to drug and other characterizations may be heterogeneous under different cell morphologies^34^,^35^. Here, the device was also used to study tumor heterogeneity, the differences on growth rate, sensitivity to indomethacin and ABCG2 protein expression level between the two types of MHCC 97L clones were further examined in this work. Firstly, we investigated the growth rate of the two kinds of clones. According to the references^36^,^37^, the cell covering area was used to calculate cell growth rate in consideration of the irregular morphologies. Type 1 MHCC 97L cells grew adherently as multilayer, we took the maximum coverage area of the type 1 MHCC 97L cells into account as the final area. The growth curves of the two clones seemed similar as shown in Fig. 5.

Actually, owe to its multilayer growth feature, the real whole area of type 1 MHCC 97L cells is larger than the area we calculated when the multilayer was supposed to spread into a monolayer like type 2 clone. Besides, the sizes of type 1 MHCC 97L and type 2 MHCC 97L are different, about 19 micrometers and 29 micrometers, respectively. According to our statistics, the average area of single type 1 MHCC 97L cell is about 692.60 μm^2^ smaller than that of type 2 MHCC 97L cell, about 1323.40 μm^2^ twice of the former. Based on the two reasons above, the actual number of type 1 MHCC 97L is much more than type 2 MHCC 97L. In conclusion, type 1 MHCC 97L grows faster than type 2 MHCC-97L.

In addition to heterogeneity study of growth rate, we further examined the clones sensitivity to indomethacin and ABCG2 protein expression level between type 1 MHCC 97L and type 2 MHCC 97L^35^,^38^,^39^,^40^.

**Figure 6:**
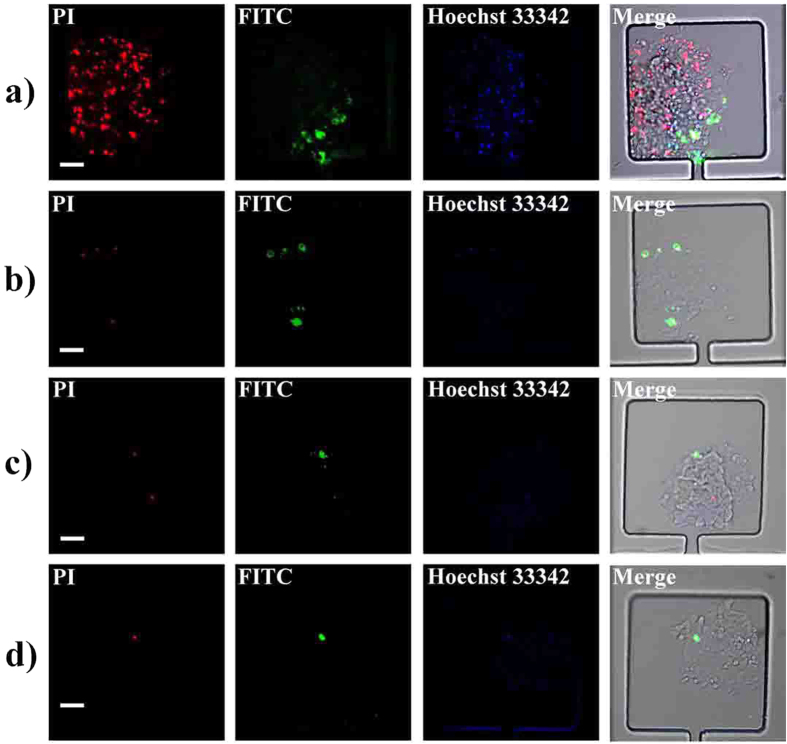
Fluorescence images of cell apoptosis under incubation with indomethacin. (**a**) type 1 MHCC 97L apoptosis images under the effect of indomethacin. (**b**) type 2 MHCC 97L apoptosis images under the effect of indomethacin. (**c**), (**d**) fluorescence images of type 1 MHCC 97L and type 2 MHCC 97L apoptosis images in control group. The red, green and blue fluorescence come from PI, FITC and Hoechst 33342, respectively. Scale bars in Figs 6a–d are all 100 μm.

According to recent report^41^, indomethacin inhibits hepatoma carcinoma cell proliferation. We here chose 0.3 mmol/L indomethacin (according to the half maximal inhibitory concentration namely IC50 experiment, Fig. S8) to examine its cytotoxicity on the two clones. An AnnexinV-FITC/PI apoptosis kit was used to confirm the cytotoxicity of indomethacin to the clones. The red and green fluorescence were respectively emitted from propidium iodide (PI) fluorescein isothiocyanate (FITC) of AnnexinV-FITC/PI apoptosis kit. Cells labeled with red fluorescence or green fluorescence were considered as apoptotic cells. Compared with the weak fluorescence intensity displayed in the group of type 2 MHCC 97L (Fig. 6b), apparent apoptosis phenomenon appeared among type 1 MHCC 97L as shown in Fig. 6a.

The results of control group were displayed in Figs 6c,d, most of the cells kept alive in the absence of indomethacin stimulation. Image-Pro Plus (IPP) was used to calculate the fluorescence intensity. The apoptosis rate was determined by the ratio of apoptotic cells to the cells labeled with Hoechst 33342. Statistic results clearly showed that the apoptosis rates of type 1 MHCC 97L and type 2 MHCC 97L were 22% and 7%, respectively (Fig. S9). The drug sensitivity results demonstrated that type 1 MHCC 97L was more sensitive to indomethacin stimulation compared with type 2 MHCC 97L.

**Figure 7:**
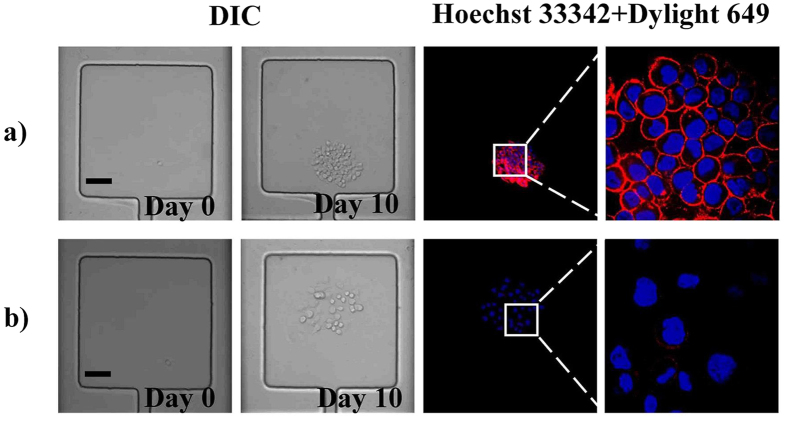
Bright field and fluorescence images of ABCG2 protein expression level of both type 1 MHCC 97L (**a**) and type 2 MHCC 97L (**b**) Scale bars in Figs 7a,b are both 100 μm.

Protein expression level is also one aspect of the cell heterogeneity. As we known, ABCG2 protein attached to ABC protein family was commonly expressed in carcinoma cell. Immunofluorescence of ABCG2 protein expression was conducted as showed in Fig. 7. The results confirmed that obvious differences of the protein expression level existed between the two clones. As shown in Figs 7a,b and Fig. S10, the ABCG2 expression level of type 1 MHCC 97L is much higher than that of type 2 MHCC 97L, which further confirmed that heterogeneity existed between the two clones.

## Discussion

In this study, a high throughput micro-chamber array device was designed for high throughput single cell isolation, long-term single cell cultivation and tumor heterogeneity studies. In comparison with the traditional single cell cloning techniques, which can only get several single cell clones through a serial of complicated and labor-intensive and reagent-consuming manipulations, the microfluidic device offered a highly efficient approach for single cell cloning and tumor heterogeneity studies. The designed micro-chamber array device has several advantages of dynamic fresh medium supplement and metabolites discharging, negligible detrimental effect of hydrodynamic shear stress on cells and high throughput single cell isolation efficiency.

Firstly, a series of condition optimizations were conducted so as to conduct the whole experiments under optimum conditions. Cell suspension concentration, flow rate as well as vacuumization time were respectively examined, we undertook the studies under 3 × 10^5^ cells/mL with a vacuumization time of half an hour with manual injection. Based on these conditions above, nearly three hundreds of clones could be obtained and could be cultured as long as for three weeks, which facilitated us to do some studies on the molecular level which were correlated with tumor heterogeneity.

Our goal was not merely restricted to single cell isolation and long-term cultivation. During the clonal cultivation course, two kinds of growth morphologies were discovered. Naturally, studies on clones growth rate, resistance to indomethacin and ABCG2 proteins expression level were respectively carried out, which were together to explore whether there were apparent tumor heterogeneity existing between the two types of clones.

The results demonstrated that long-term single cell cultivation could be realized in the lateral chambers and several hundreds of single cell clones could be obtained in one device, which greatly benefit tumor heterogeneity study at single cell clone level. As a proof of concept, a series of heterogeneity researches on MHCC-97L cell line, such as cell morphology, growth rate, clones resistance to indomethacin and ABCG2 protein expression level have been carried out in the device. In the future work, we anticipate that the high integration chamber array will pave ways for drug screening, tumor biomarker discovering and tumor metastasis studies under mild fabrications and versatile function elements installation.

## Methods

### Device design and fabrication

On the basis of our previous work^42^, we designed a lateral chambers array. The designed device contains 1400 lateral square chambers with 500 micrometers per side, the width of the lateral entrance and main channel are 30 micrometers and 100 micrometers, respectively. The device sketch is displayed in Figs 1a,c. High resolution laser printer was used to print the designed structure on a plastic film. Then, we took use of negative photoresist SU-8 2050 (Clariant Corporation, USA) to fabricate a 50 micrometers height channel through soft-lithography technique. Series of procedures including spin coating, bake, exposure and development were used to fabricate the mold. After that, the manufactured silicon wafer (Siltronix SAS, France) was then exposed to trimethylchlorosilane vapor for 2 minutes to avoid the adhesion between the wafer surface and PDMS (GE Toshiba silicones Co. Ltd Shizuoka, Japan). Then, the mixture of PDMS and its curing agent were poured onto the surface of the silicon wafer, then, placed in oven under 75 °C for about 3 hours. Cured PDMS was peeled from the silicon wafer and access holes for inlet and outlet were punched with metal pipes. PDMS and cover glass were irreversibly bonded together after two minutes oxygen plasma treatment. Then, the device was washed with ultrapure water and put in an aluminum container for sterilizing in an autoclave for half an hour at 120 °C until use.

### Cell culture and maintenance

MHCC 97L cell is hepatocellular carcinoma cell line obtained from Zhongnan Hospital of Wuhan University, China. Cells were maintained through passaging with 0.25% Trypsin–EDTA (Sigma) three times a week with Roswell Park Memorial Institute (RPMI) 1640 medium (Gibco) supplemented with 10% fetal bovine serum (FBS, Perbio) and 1% penicillin/streptomycin (Gibco) at 37 °C under 5% CO_2_ atmosphere.

### Cell self-loading and on-chip single cell clonal cultivation

The sterile device was put in a vacuum vessel for half an hour to form negative pressure surroundings. MHCC 97L cells were detached with 0.25% Trypsin–EDTA and suspended in fresh RPMI 1640 medium supplemented with 10% FBS. The cell suspension was under shake for nearly 30 seconds and then manually injected into the degassed device through inlet with a 1 mL syringe. Once the main channel was filled with cell suspension, the chambers could be filled in less than 5 minutes. Then, another 10 mL syringe containing fresh RPMI 1640 medium was used to wash away the unloaded cells in the main channel for 30 seconds. Finally, the device was connected with a syringe pump (Harvard Apparatus, PHD 2000) through pipe and observed under a microscope. The chambers that loaded a single cell were marked for further single cell clonal cultivation and subsequent tumor heterogeneity research. Then, the device was dynamically perfused with fresh RPMI 1640 medium under a flow rate of 20 μL/h and maintained in the incubator at 37 °C with 5% CO_2_ for single cell clonal proliferation and long-term cultivation.

### Clone sensitivity to indomethacin

MHCC 97L cells were cultured in the device up to day 11. Then, the clones were incubated with 0.3 mmol/L indomethacin (Sigma) diluted with RPMI 1640 medium and cultured up to day 14. RPMI 1640 medium without indomethacin was used as the control groups. On the day 14, AnnexinV-FITC/PI apoptosis kit (Sigma) was used to identify chemosensitivity of the clones to indomethacin. The results were observed and imaged under a spinning-disk confocal microscope (Andor Revolution XD) equipped with an Olympus IX 81 microscope, a Nipkow disk type confocal unit (CSU 22, Yokogawa), a CO_2_ online culture system (INUBG2-PI), and an EMCCD (Andor iXon DV885K).

### Clone ABCG2 protein expression level

MHCC 97L cells were cultured in the device up to day 10. Immune fluorescence analysis of subcellular ABCG2 protein expression level was examined. Firstly, the clones were fixed with 4% (w/v) paraformaldehyde for 3 h at room temperature (RT) and washed with phosphate buffered solution (PBS, pH 7.4). Secondly, the clones were permeabilized with 0.01% Triton X-100 for 2 h at RT and then washed with PBS. Then, the clones were incubated with mouse monoclonal primary antibody (5D3) against ABCG2 (SantaCruz) under optimal dilution at 37 °C for 4 h and washed with PBS. Finally, dylight 649-conjugated goat anti-mouse IgG (Earthox) was used to incubate with the clones at 37 °C for 3 h and washed with PBS. All the solutions in the immunofluorescence analysis were actuated by the syringe pumps under a flow rate of 50 μL/h. The immune fluorescence was observed and photographed under a spinning-disk confocal microscope.

## Additional Information

**How to cite this article**: Shen, F.-M. *et al.* A High Throughput Micro-Chamber Array Device for Single Cell Clonal Cultivation and Tumor Heterogeneity Analysis. *Sci. Rep.* **5**, 11937; doi: 10.1038/srep11937 (2015).

## Supplementary Material

Supplementary Information

Supplementary Movie
