# Supplementary material for: A High Throughput Micro-Chamber Array Device for Single Cell Clonal Cultivation and Tumor Heterogeneity Analysis
Source: Sci Rep. 2015 Jul 7;5:11937. doi: 10.1038/srep11937 (PMC4493670; doi:10.1038/srep11937)
Supplement: Supplementary Information [file srep11937-s1.pdf]

Electronic Supplementary Material

A High Throughput Micro-Chamber Array Device for Single Cell Clonal Cultivation and Tumor Heterogeneity Analysis

Feng-Min Shen,† Lian Zhu,† Heng Ye, Yu-Jun Yang, Dai-Wen Pang, Zhi-Ling Zhang*

Key Laboratory of Analytical Chemistry for Biology and Medicine (Ministry of Education), College of Chemistry and Molecular Sciences, Wuhan University, Wuhan, 430072, P. R. China.;

* Corresponding author: E-mail: zlzhang@whu.edu.cn. Fax: +86-27-68754067; Tel: +86-27-68756759

† These authors contributed equally to this work.

COMSOL Multiphysics 4.3 simulation. In order to demonstrate the detrimental impacts of hydrodynamic shear stress on the isolated single cells within the lateral chambers was negligible, we used COMSOL Multiphysics 4.3 to simulate the surface velocity field, results revealed that the surface velocity within the lateral chambers was very low (shown with blue color in Fig. S1). According to the reports, surface velocity has a positive correlation with hydrodynamic shear stress, so, we believed that our designed device could effectively decrease the hydrodynamic shear stress on the isolated single cells within the lateral chambers, which guaranteed long-term clonal cultivation and tumor heterogeneity studies.

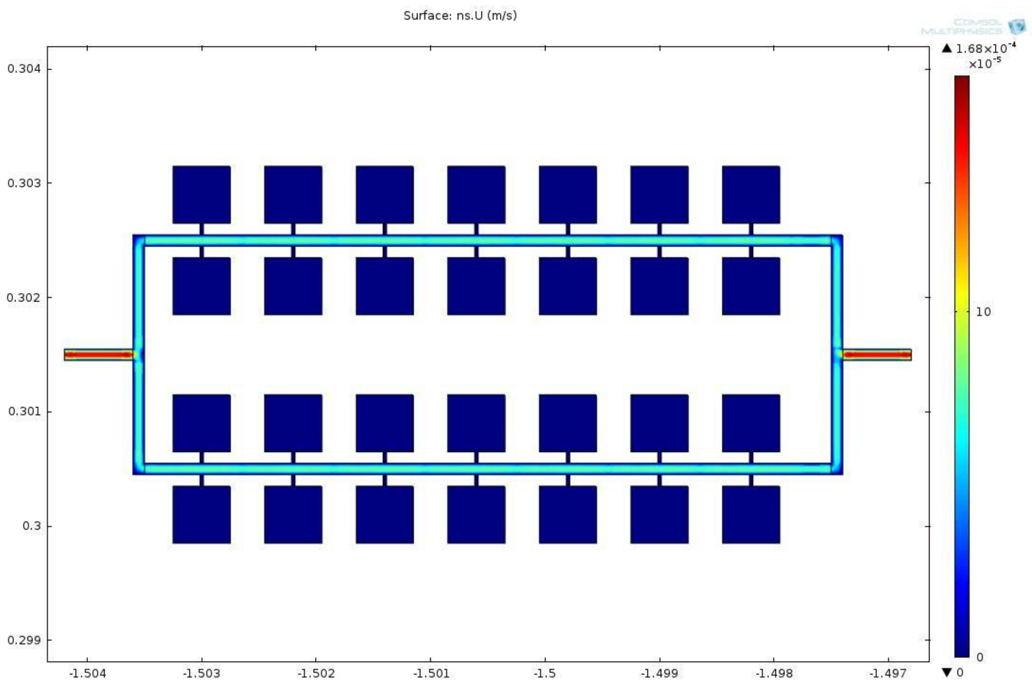

Fig. S1 Simulation of the surface velocity fields of the designed chip

Red ink diffusion simulation. Effective diffusion between main channels and micro-chambers was essential for long-term single cell clonal cultivation attributed to the adequate fresh medium supplement and poisonous excretion discharging. Here, we utilized red ink diffusion to explain the diffusion between the two units as shown in Fig. S2a, b.

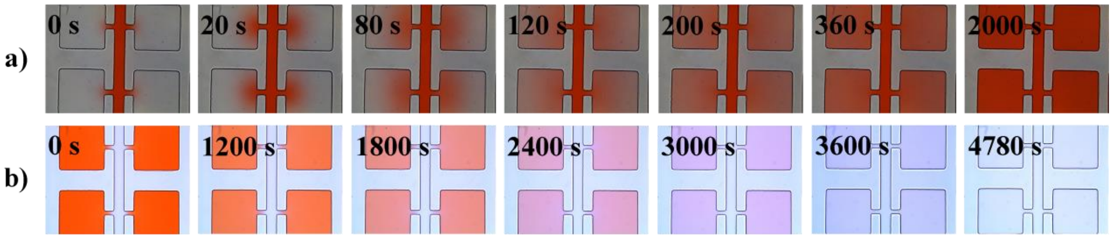

Fig. S2 Red ink diffusion into and out of the chip. a) red ink could effectively diffuse into the lateral chambers in about half an hour. b) red ink diffused from the lateral chambers into the main channel.

Injection rate and vacuumization time. The influence of suspension flow rate on single cell isolation efficiency was aslo investigated. From 50 to 1000 μL/min, the results showed that injection rate hardly have influence on single cell isolation efficiency (Fig. S3a).

Negative pressure inside the chamber may also influence cell suspension loading into lateral chambers. The following mathematical expression explained the relationship between absolute pressure inside micro-container and time for vacuumization. From 2 minutes to 40 minutes, we examined six points to determine an optimum time. It proved that half an hour was

enough as shown in Fig. S3b.

$$\mathbf{P} = K_3 + K_1 \mathbf{exp}(-K_2 \mathbf{T})$$

P: Pressure inside micro-container (Absolute vacuum)

T: Vacuumization time

K_3: Ultimate vacuum of pump

K_1, K_2: Relative constants with pump, scale of micro-container and ambient pressure

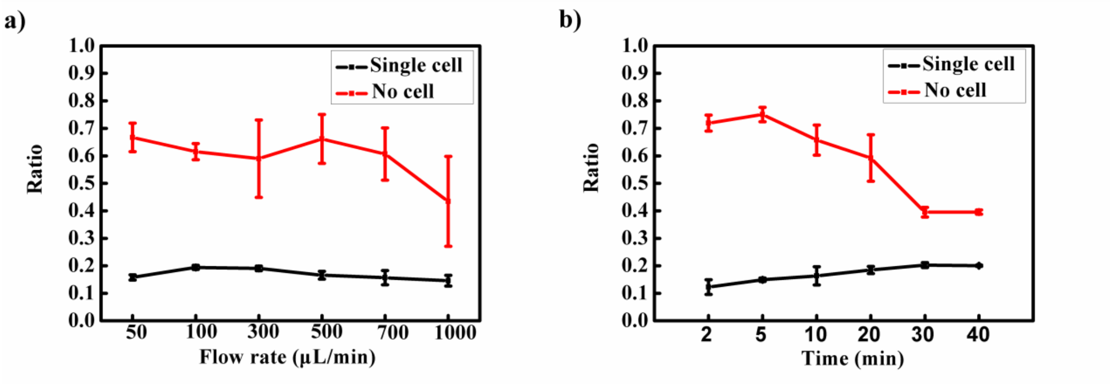

Fig. S3 Injection flow rate and vacuumization time optimization. a) relationship between suspension flow rate and single cell isolation efficiency. b) relationship between time for vacuumization and single cell isolation efficiency.

Long-term clonal cultivation. The device we designed deserved the following advantages: enough space for long-term clonal cultivation, continuously dynamic perfusion for nutrients supplements and poisonous metabolizes discharging, and negligible hydrodynamic shear stress. Clonal cultivation was realized for as long as 19 days as shown in Fig. S4.

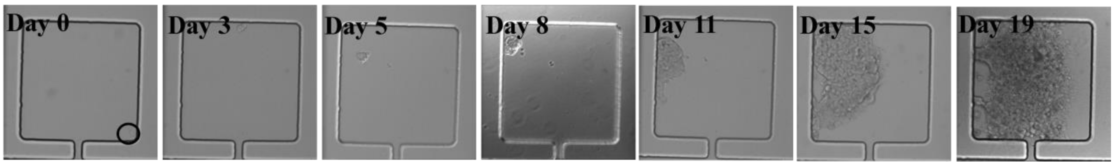

Fig. S4 Long-term single cell clonal cultivation from day 0 to day 19, the circle refers to single cell.

Jurkat T cell clonal cultivation. In order to confirm whether our designed device was apt for other kinds of tumor cells isolation and clonal proliferation, Jurkat T cell was chosen to complete the task as shown in Fig. S5.

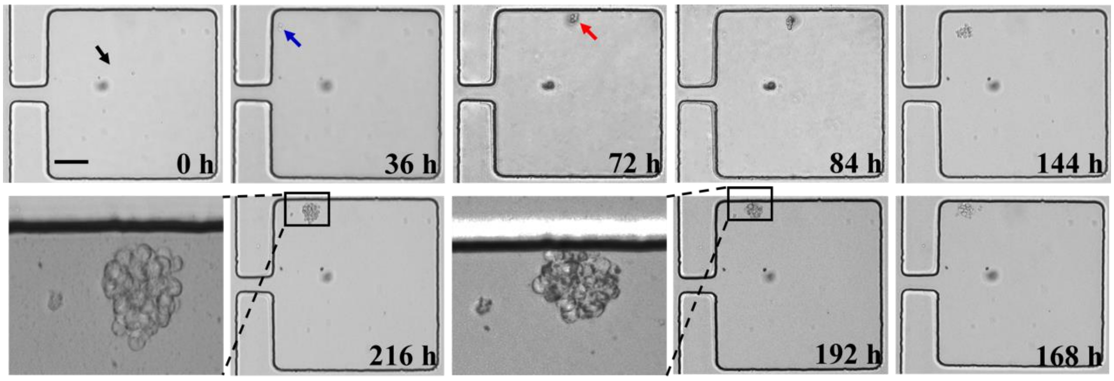

Fig. S5 Long term Jurkat T cell clonal cultivation from 0 h to 216 h, black, blue and red arrow are referred to single cell, two cells and four cells respectively. Scale bar referred to 100 μm.

Suspension cell clonal cultivation. In general, most of the researches were focused on adherent cell cultivation. We here tried to cultivate suspension cell with this device, the results demonstrated that as long as one week cultivation could be accompanied (Fig. S6).

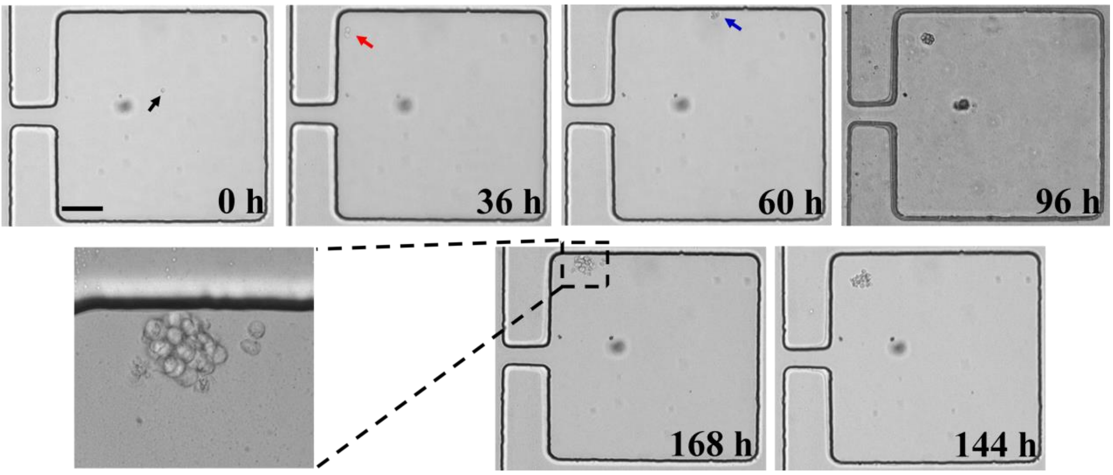

Fig. S6 Long term suspension cell clonal cultivation from 0 h to 168 h, black, red and blue arrow are referred to single cell, two cells and four cells respectively. Scale bar referred to 100 μm.

Ratios of type 1 and type 2 MHCC-97L cell. Two kinds of morphologies namely type 1 and type 2 MHCC-97L cells were discovered during the clonal cultivation. Nearly hundreds of clones were formed once, we here examined the ratio of the two clones (Fig. S7).

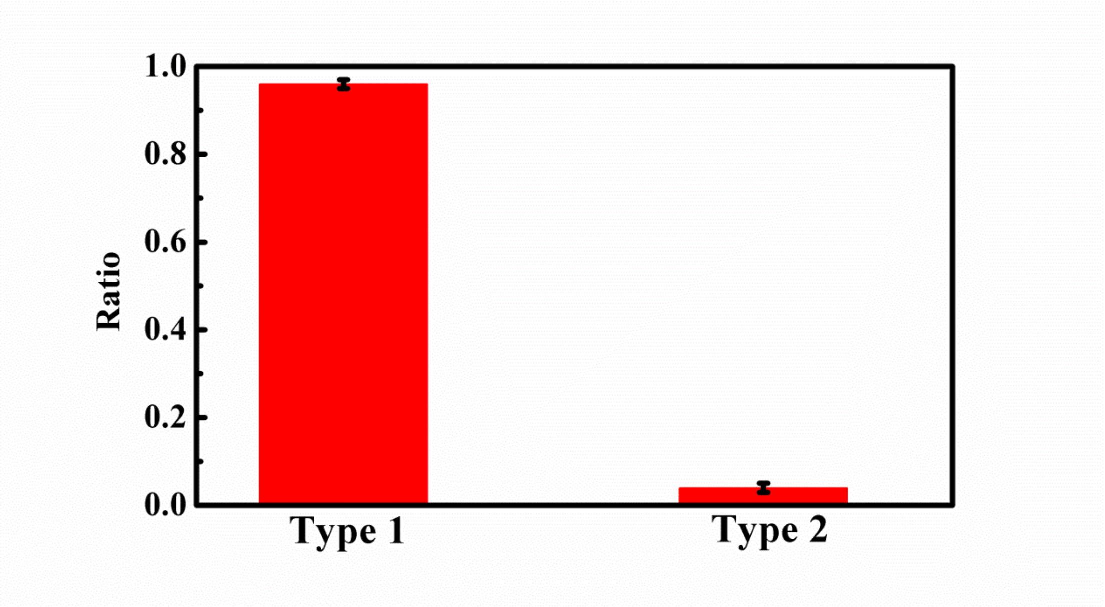

Fig. S7 Ratios of type 1and type 2 MHCC-97L cells to the whole clones

Indomethacin concentration optimization. Before incubated the clones with indomethacin, optimum drug concentration was examined based on MTT assay. Firstly, we chose the following concentrations 0.05 mmol/L, 0.1 mmol/L, 0.2 mmol/L and 0.5 mmol/L, apoptosis ratios to hepatoma carcinoma cell MHCC 97L were respectively recorded at 24 h, 48 h and 72 h. We calculated the IC50 at 72 hour and determined 0.3 mmol/L as the final concentration as showed in Fig. S8.

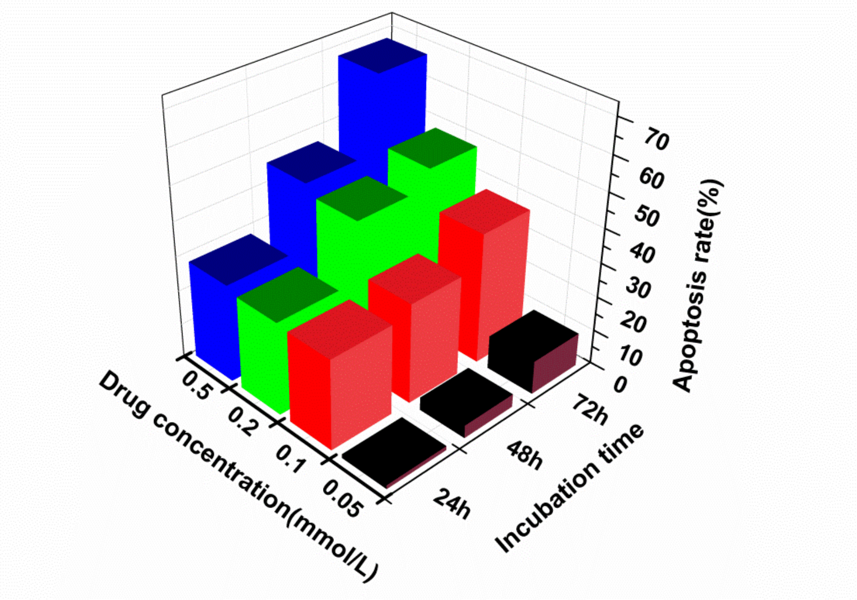

Fig.S8 MTT assay for determination an optimum indomethacin concentration.

$$\mathbf{lgIC50 = Xm - I\left(P - \frac{3 - Pm - Pn}{4}\right)}$$

X_m: lg maximum dose

I: lg (maximum dose/ adjacent dose)

P: Sum of reaction rate of the experimental group

P_m: Maximum reaction rate of experimental group

P_n: Minimum reaction rate of experimental group

Clone sensitivity to indomethacin. Both of the two kinds of clones were incubated with indomethacin at day 11, three days later, the apoptosis rate were evaluated through the fluorescence intensity of dye derived from the AnnexinV-FITC/PI apoptosis kit (Fig. S9). The following statistics are corresponding to the confocal images in the main text.

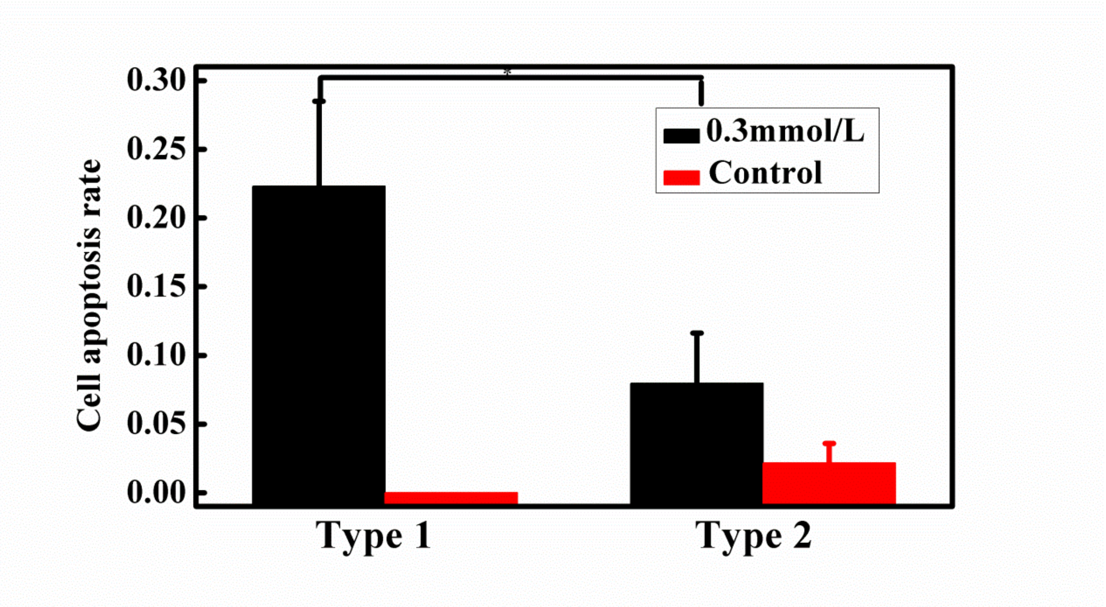

Fig. S9 Apoptosis rate of type 1 and type 2 MHCC-97L cells after incubated with indomethacin both in experimental group and control group. (All experiments were triplicated and error bars represent the mean±SD.*P < 0.05 vs control, n =3.)

Clone ABCG2 protein expression level. ABCG2 protein（ATP-binding cassette, subfamily G, member 2）as the member of ABC protein family. ABCG2 was commonly expressed in liver tissues, but the mechanisms of their resistance to anti-cancer drugs remained unknown. Immune fluorescence technique was utilized to qualitatively characterize ABCG2 protein expression level, then, fluorescence intensity was calculated through IPP 6.0 as shown in Fig. S10.

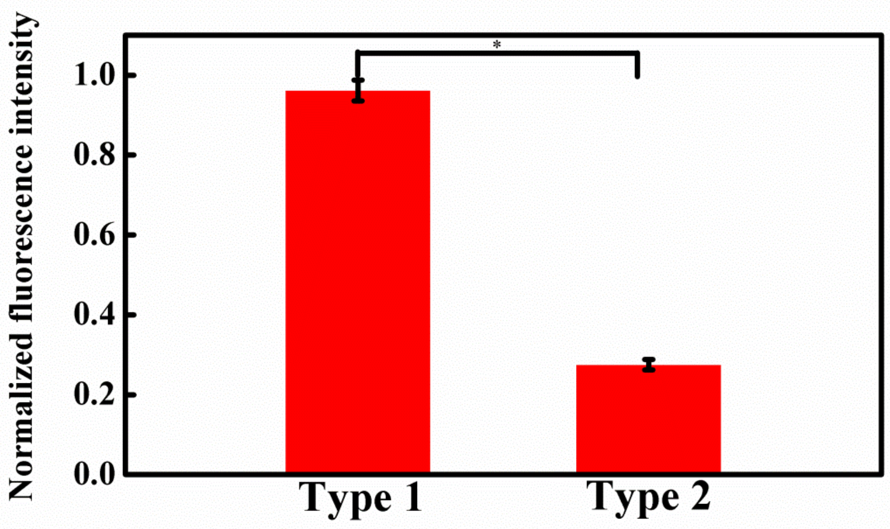

Fig. S10 Statistics of the fluorescence intensity of ABCG2 protein expression of both type 1 and type 2 MHCC-97L cells (All experiments were triplicated and error bars represent the mean±SD.*P < 0.05, n =3.)

Fig. S1 Movie The movie depicts the dynamic course of single cell loading into the lateral chamber.
